# Chidamide and orelabrutinib synergistically induce cell cycle arrest and apoptosis in diffuse large B-cell lymphoma by regulating the PI3K/AKT/mTOR pathway

**DOI:** 10.1007/s00432-024-05615-7

**Published:** 2024-02-21

**Authors:** Chunyan Wu, Shilv Chen, Zhimin Wu, Jiao Xue, Wen Zhang, Shan Wang, Xindong Zhao, Shaoling Wu

**Affiliations:** 1 Department of Hematology, The Affiliated Hospital of Qingdao University, No. 16 Jiangsu Road, Qingdao, 266003 Shandong China; 2 Department of Medicine, Qingdao University, Qingdao, China

**Keywords:** Chidamide, Orelabrutinib, Cell cycle arrest, Apoptosis, Diffuse large B-cell lymphoma, PI3K/AKT/mTOR pathway

## Abstract

**Objective:**

The initial therapeutic approach for diffuse large B-cell lymphoma (DLBCL) entails a rituximab, cyclophosphamide, doxorubicin, vincristine, and prednisone (R-CHOP) regimen. However, 40% of patients exhibit suboptimal responses, with some experiencing relapse and refractory conditions. This study aimed to explore novel therapeutic strategies and elucidate their underlying mechanisms in DLBCL.

**Methods:**

Bioinformatics techniques were employed to scrutinize correlations between the HDAC1, HDAC2, HDAC3, HDAC10, BTK, MYC, TP53, and BCL2 genes in DLBCL. In vitro experiments were conducted using DB and SU-DHL-4 cells treated with chidamide, orelabrutinib, and a combination of both. Cell viability was assessed by cell counting kit-8. Cell apoptosis and the cell cycle were determined using flow cytometry. Reactive oxygen species (ROS) production and mitochondrial function were assessed through ROS and JC-1 staining. RNA sequencing and western blot analyses were conducted to elucidate the molecular mechanisms underlying the combined action of chidamide and orelabrutinib in DLBCL cells.

**Results:**

This investigation revealed markedly enhanced antiproliferative effects when chidamide was combined with orelabrutinib. Compusyn software analysis indicated a synergistic effect of chidamide and orelabrutinib in inhibiting DLBCL cell proliferation, with a combination index (CI) < 1. This synergy further manifested as augmented cell cycle arrest, apoptosis induction, the downregulation of cell cycle-associated and antiapoptotic proteins, and the upregulation of proapoptotic proteins. Furthermore, the western blot and RNA-Seq findings suggested that combining chidamide and orelabrutinib modulated the PI3K/AKT/mTOR signaling pathway, thereby promoting DLBCL cell cycle arrest and apoptosis.

**Conclusion:**

The findings of this study provide a compelling justification for the clinical utilization of chidamide and orelabrutinib to treat relapsed/refractory DLBCL.

**Supplementary Information:**

The online version contains supplementary material available at 10.1007/s00432-024-05615-7.

## Introduction

Diffuse large B-cell lymphoma (DLBCL) is a heterogeneous and aggressive non-Hodgkin’s lymphoma subtype. Over 60% of DLBCL patients can be cured with R-CHOP (rituximab, cyclophosphamide, doxorubicin, vincristine, and prednisone) immunochemotherapy; However, those who do not respond to R-CHOP often have worse outcomes, including relapsed or refractory disease, which are fatal in most patients (Fan et al. 2017; Sehn and Salles 2021).

The World Health Organization (WHO) classifies DLBCL with MYC and BCL2 rearrangements and the simultaneous occurrence of BCL6 gene rearrangement as double-hit lymphoma (DHL) or triple-hit lymphoma (THL). These subtypes are characterized by a rapidly progressing clinical course that is refractory to aggressive treatment and associated with a short survival duration (Green et al. 2012). Double-expression lymphoma (DEL), which is DLBCL that coexpresses MYC (immunohistochemical threshold ≥ 40%) and BCL2 (immunohistochemical threshold ≥ 50%) proteins as determined via immunohistochemistry (IHC), accounts for 21%–34% of patients with newly diagnosed DLBCL (Herrera et al. 2017; Riedell and Smith 2018). DEL patients also demonstrate an inferior prognosis after R-CHOP induction therapy, similar to that of DHL patients (Hu et al. 2013; Herrera et al. 2017). Thus, current treatments are insufficiently effective, and the development of new targeted therapies is imperative.

Epigenetic alterations have been found to contribute to lymphoma growth and the generation of chemoresistant phenotypes. Chidamide is a novel inhibitor of histone deacetylases (HDACs) that selectively inhibits HDAC1, 2, 3, and 10 and has been marketed in China for treating peripheral T-cell lymphoma (PTCL) since 2015 (Moskowitz and Horwitz 2017; Liang et al. 2021). Chidamide can induce growth arrest and apoptosis in blood and lymphoid tumor cells. Treatment with chidamide as a single agent or in combination with a second agent is being investigated in preclinical and clinical trials for certain solid and hematologic tumors. Chidamide has been clinically observed to be effective in patients with relapsed/refractory B-cell lymphoma (Li et al. 2019); however, its mechanism of action is not clear. Chidamide inhibits Myc and BCL2 in acute myeloid leukemia (Li et al. 2015), and its combination with doxorubicin in treating T-cell lymphoma also downregulates BCL2 (Zhang et al. 2017). Clinical trials have demonstrated that the outcome of DEL patients treated with R-CHOP is significantly worse than that of non-DEL patients; However, no significant difference was observed in patients treated with chidamide combined with R-CHOP (Zhang et al. 2020). Thus, chidamide may improve treatment outcomes in patients with DLBCL by targeting MYC and BCL2 (Luo et al. 2022). Experimental results have shown that chidamide could increase the acetylation level of histone H3 and H4 and caspase-3 activity in B-cell lymphoma cells, induce lymphoma cell cycle arrest, and promote cell apoptosis (Li et al. 2012). Furthermore, chidamide can restore the drug sensitivity of resistant tumor cells through epigenetic regulatory mechanisms (Camicia et al. 2015).

Bruton’s tyrosine kinase (BTK) is a key kinase in the B-cell surface antigen (BCR) signaling pathway, which plays an important role in B-cell growth, development, and differentiation, aberrant BTK function is thought to be associated with cancer and autoimmune diseases (Liu et al. 2020). BTK inhibitors can transmit cytokine receptor and BCR signaling pathway signals and inhibit the proliferation and growth of malignant B cells. Orelabrutinib, a BTK inhibitor developed by InnoCare Pharma, was approved by the National Medical Products Administration (NMPA) in December 2020 for treating mantle cell lymphoma (MCL), chronic lymphocytic leukemia (CLL), and small lymphocytic leukemia (SLL). The combination of orelabrutinib and rituximab could preserve rituximab-induced NK cell-mediated antibody-dependent cellular cytotoxicity (ADCC) and enhance tumor cell apoptosis in vitro (Yu et al. 2021). Orelabrutinib combined with venetoclax synergistically could induce DHL cell death and cell cycle arrest, as well as inhibit cell proliferation (Pan et al. 2023). A once-daily orelabrutinib dosing regimen achieves sustained BTK occupancy at 24 h (Dhillon 2021). An orelabrutinib-based regimen with an objective response rate (ORR) of 60.00% showed encouraging anticancer results in patients with relapsed/refractory (R/R) nerve system lymphoma (CNSL) (Wu et al. 2022).

In modern medicine, there is an urgent need to explore novel biologics and targeted therapies that employ a “one plus one is greater than two” combination approach. Such therapeutic strategies can potentially enhance efficacy beyond that of monotherapy, consequently improving the overall prognosis. This study explored the molecular mechanism and potential antitumor effect of chidamide combined with orelabrutinib in DLBCL and provided new ideas and research evidence for developing more effective DLBCL treatment regimens.

## Materials and methods

### Data acquisition and processing

The Cancer Genome Atlas (TCGA) database (https://portal.gdc.cancer.gov) was used to obtain data from 47 lymph node samples from DLBCL patients, while the Genotype-Tissue Expression (GTEx) database (https://commonfund.nih.gov/GTEx) was used to obtain data from 337 healthy subjects.

### cBioPortal

cBioPortal, a straightforward web interface, was utilized to analyze gene variation in DLBCL, including amplification, mutation, and copy number variation (http://www.cbioportal.org/). An overview of genetic modifications was also obtained to visualize all aspects of each mutation category in each sample.

### Gene expression profiling interactive analysis

The Gene Expression Profiling Interactive Analysis (GEPIA) database is a comprehensive cancer genomics dataset that combines extensive data from TCGA and GTEx. We employed GEPIA (accessible at http://gepia.cancer-pku.cn/) to assess variations in gene expression and correlations between two genes in DLBCL and normal tissues using analysis of variance (ANOVA).

### Reagent

Chidamide and orelabrutinib were kindly provided by Chipscreen Biosciences (Shenzhen, China) and InnoCare Pharma Limited (Beijing, China), respectively. The agents were dissolved in dimethyl sulfoxide (DMSO, Sigma, USA) to obtain stock solutions of 50 and 150 mM. The stock solutions were stored at − 80 °C.

### Cell lines and culture

Human B-lymphoma cell lines DB (MYC/BCL2 rearranged DHL cells with mutant (MUT)-TP53) and SU-DHL-4 (MYC/BCL2-positive DLBCL cells with wild-type (WT)-TP53) were obtained from Procell Life (Wuhan, China). DB and SU-DHL-4 cells were maintained in Roswell Park Memorial Institute-1640 (RPMI-1640; Procell, Wuhan, China) supplemented with 10% fetal bovine serum (FBS; Procell, Wuhan, China). The medium was supplemented with 100 U/ml penicillin and 100 mg/ml streptomycin, and the cells were incubated at 37 °C in a humidified 5% CO2 atmosphere.

### Cell viability assay

Cells were inoculated into 96-well plates and treated with the designated drug, and cell counting kit-8 (CCK‑8; Yeasen, Shanghai, China) reagent was added after 24 or 48 h of action. Absorbance was detected at 450 nm on a microplate absorbance reader (Tecan Group Ltd., Switzerland).

### Cell apoptosis assay

The cells were inoculated into six-well plates and treated with various drug doses for 48 h. Cells were harvested and washed twice with PBS precooled at 4 °C. Cells were resuspended in 100 µl binding buffer. Subsequently, 5 µl Annexin V-FITC and 10 µl propidium iodide (Yeasen, Shanghai, China) were added to the cell suspension, which was gently mixed and reacted in the dark for 15 min, followed by 300 µl binding buffer. Using flow cytometry cell apoptosis was examined within 1 h.

### Cell cycle assay

Propidium iodide (PI) is a fluorescent dye that can be used in conjunction with double-stranded DNA to detect the cell cycle. The cells were plated onto six-well plates and treated with various drug doses for 48 h. Cell samples should be rinsed once with precooled PBS, centrifuged, gently mixed with 70% ethanol, and fixed at 4 °C for the night to prepare them for future use. The cell samples were stained using with a staining solution containing PI and RNase A (Beyotime, Shanghai, China) and incubated for 30 min at 37 °C in the dark. The samples were stored at 4 °C and detected by flow cytometry within 24 h.

### Measuring the reactive oxygen species (ROS) levels inside cells

The cells were plated onto six-well plates and treated with various drug doses for 48 h. Following treatment, the cells were incubated with DCFH-DA for 20 min at 37 °C and examined under a fluorescence microscope.

### Mitochondrial membrane potential (MMP) measurement by JC-1 staining

After exposure to various drug doses, the cells were collected, resuspended in RPMI 1640 medium, treated with JC-1 (Yeasen, Shanghai, China) at ambient temperature for 20 min, and analyzed through fluorescence microscopy.

### RNA sequencing analysis

DB cells were treated with drugs for 48 h and then divided into control, chidamide single-drug, orelabrutinib single-drug, and combination groups. *P* < 0.05 was used to determine the significance of the differentially expressed genes (DEGs). RNA-seq was performed using the Illumina platform and analyzed by ANNOROAD (c.solargenomics.com). DESeq 2 was used for differential gene expression analysis. Genes with a fold change ≥ 2 and *q* < 0.05 were considered significant DEGs. Gene Ontology (GO) enrichment analysis of the DEGs was implemented using clusterProfiler packages based on Wallenius' noncentral hypergeometric distribution. We used clusterProfiler software to test the statistical enrichment of DEGs in Kyoto Encyclopedia of Genes and Genomes (KEGG) pathways.

### Western blot analysis

Cells were collected, washed twice with PBS, and lysed for 30 min at 4 °C in RIPA buffer (Beyotime, Chengdu, China). The lysate was centrifuged at 12,000×*g* for 15 min at 4 °C, and the protein content was assessed using a BCA assay (Elabscience, Wuhan, China). An equal amount of protein was separated by sodium dodecyl sulfate–polyacrylamide gel electrophoresis (SDS–PAGE) and transferred to a polyvinylidene difluoride (PVDF) membrane. The PVDF membrane was then hermetically sealed with 5% (0.05 g/mL) skim milk, incubated overnight at 4 °C with the primary antibody, washed, and incubated with a secondary antibody for 70 min. The membranes were then cut horizontally. Finally, protein detection was conducted using a chemiluminescence substrate.

### Statistical analysis

All experiments were performed at least three times. Data were analyzed using GraphPad Prism 9.3.1 (GraphPad Software, USA) and expressed as the mean ± standard deviation of at least three experiments. One-way ANOVA was used to compare the cell viability and proliferation results and the different time points and concentrations in DB and SU-DHL-4 cells. P < 0.05 was considered to indicate significance.

## Results

### Correlation between BCL2, c-Myc, TP53, HDAC1, HDAC2, HDAC3, HDAC10*,* and BTK expression in DLBCL

**Fig. 1:**
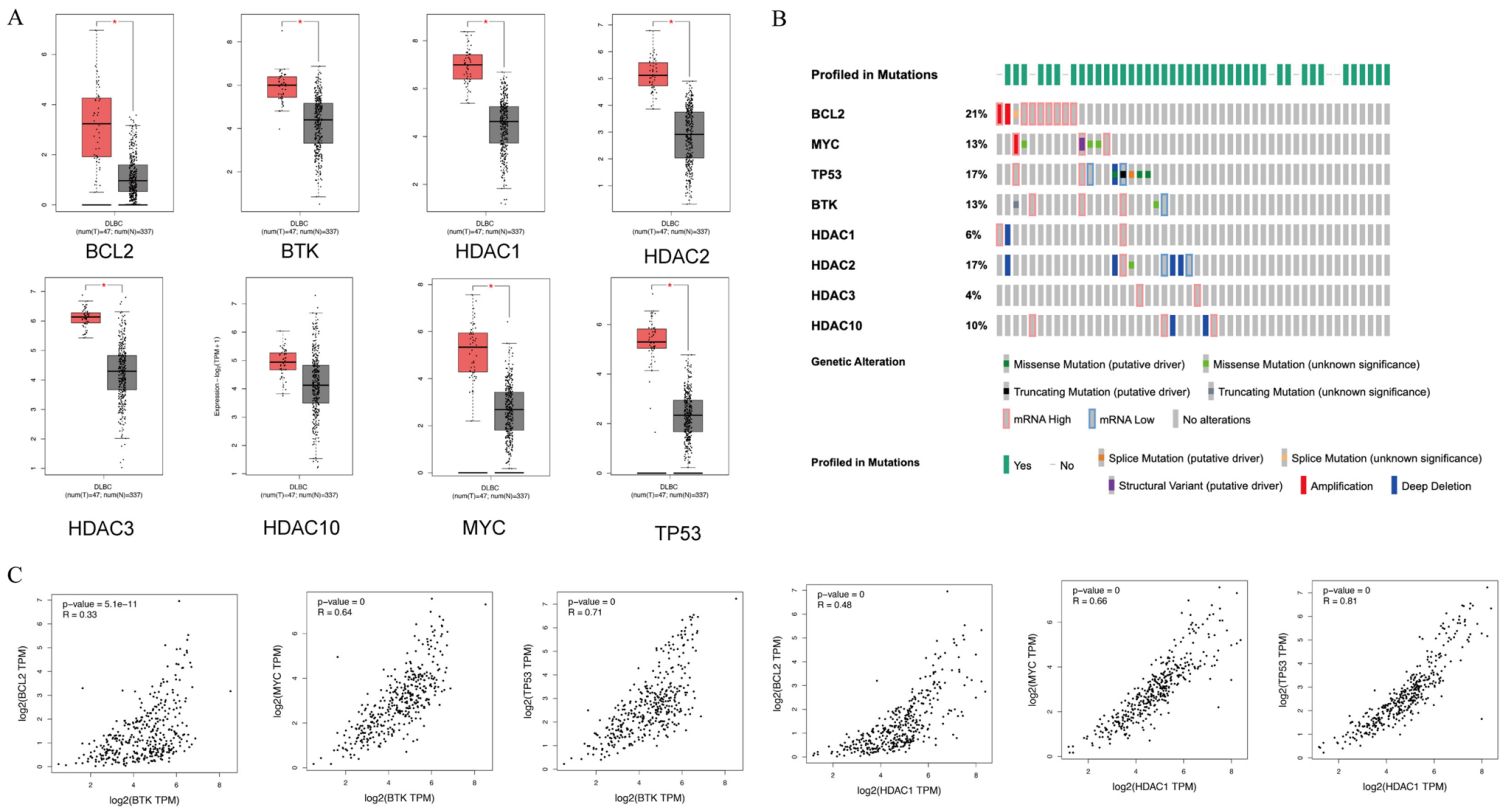
**A** Box plots show the differential expression of BCL2, MYC, TP53, BTK HDAC1, HDAC2, HDAC3, and HDAC10 in DLBCL. **B** The mutation rates of these eight genes in DLBCL. **C** The first three plots are correlation analyses of BTK with BCL2, MYC, and TP53, respectively. The last three plots are correlation analyses of HDAC with BCL2, MYC, and TP53, respectively. (*P < 0.05)

The GEPIA database was used to determine the mRNA expression levels of BCL2, c-Myc, TP53, HDAC1, HDAC2, HDAC3, HDAC10, and BTK in DLBCL. Forty-seven lymph node samples from patients with DLBCL and 337 whole-blood-cell samples were included. Compared with normal tissues, in DLBCL, the expression levels of BCL2, Myc, TP53, HDAC1, HDAC2, HDAC3, and BTK were significantly elevated (Fig. 1A, P < 0.05). cBioPortal revealed high mutation rates in BCL2, Myc, TP53, HDAC1, HDAC2, HDAC3, HDAC10, and BTK in the DLBCL samples (Fig. 1B). HDAC1 has been well characterized and may indicate a poor prognosis in DLBCL cases (Min et al. 2012), so we performed a correlation analysis of HDAC1, BTK, BCL2, TP53, and MYC. The correlation analysis demonstrated that BTK strongly correlated with BCL2 (Fig. 1C).

### Effects of chidamide and orelabrutinib on the proliferation of DLBCL cells

**Fig. 2:**
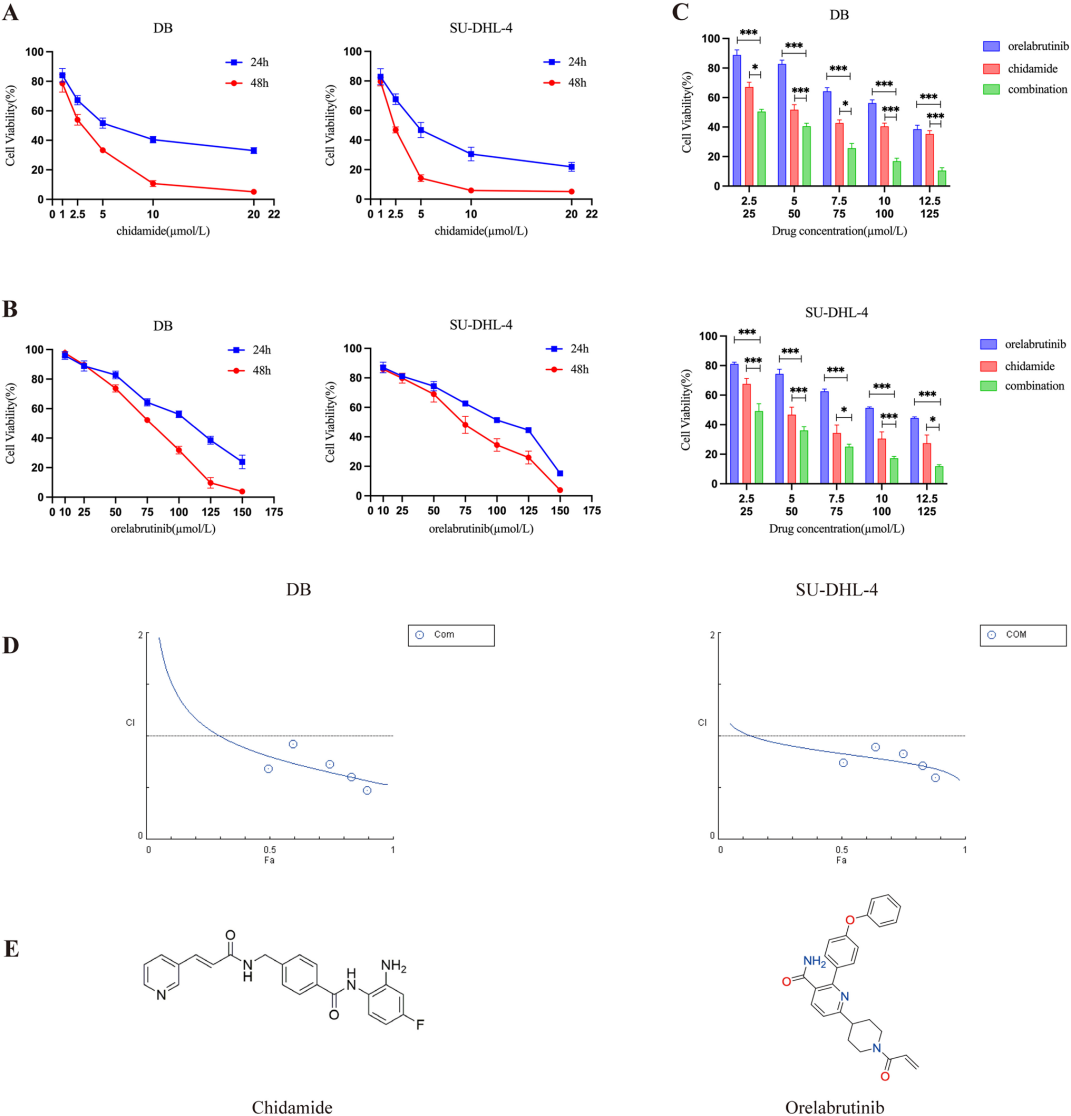
**A** and **B** Chidamide (0, 0.5, 1, 2.5, 5, 10, 20 µmol/L) or orelabrutinib (0, 10, 25, 50, 75, 100, 125, 150 µmol/L) were used to treat SU-DHL-4 and DB cells for 24 and 48 h, respectively. **C**, **D** SU-DHL-4 cells and DB cells were treated with the combination or a single drug for 24 h. The cell viability of the combination group was lower than that of the single drug group. The combination index (CI) calculated by Compusyn software showed that the CI values of the corresponding concentrations were all below 1, indicating that the combination of the two drugs had a synergistic effect. Additive effect (CI = 1), synergism (CI < 1), and antagonism (CI > 1). E. Chemical structures of chidamide and orelabrutinib. (*P < 0.05, ***P < 0.001)

First, we investigated the sensitivity of DLBCL cell lines DB and SU-DHL-4 to chidamide and orelabrutinib. A CCK-8 assay was then used to assess cell viability. When exposed to a range of specified concentrations of chidamide and orelabrutinib for 24 and 48 h, the proliferation rates of both cell lines were significantly reduced in a dose- and time-dependent manner (Fig. 2A and B). Next, we used the Chou–Talalay method to assess the effects of the drug combinations on SU-DHL-4 and DB cells over 24 h (Fig. 2C). With multiple concentration points above or below the 24 h IC_50_ value (Table 1), we set constant ratio drug combinations. Chidamide and orelabrutinib together were found to have a greater inhibitory impact than either medication alone. The Chou–Talalay (Chou 2010) approach was used to visually examine the combination index (CI) value (Fig. 2D). The CI values for the SU-DHL-4 and DB cell lines were 0.83 and 0.80, respectively, at 50% cell proliferation (fraction affected (Fa) = 0.5), indicating substantial synergism (CI < 1) of the two drugs.

**Table 1:** IC50 values calculated with linear regression based on CCK-8 data

Cell line	IC50 value (µmol/L), 24 h	
	Chidamide	Orelabrutinib
DB	6.492	100.3
SU-DHL-4	4.769	91.21

### Effects of the combination of chidamide and orelabrutinib in DLBCL cell apoptosis and cell cycle at the G0/G1 phase

**Fig. 3:**
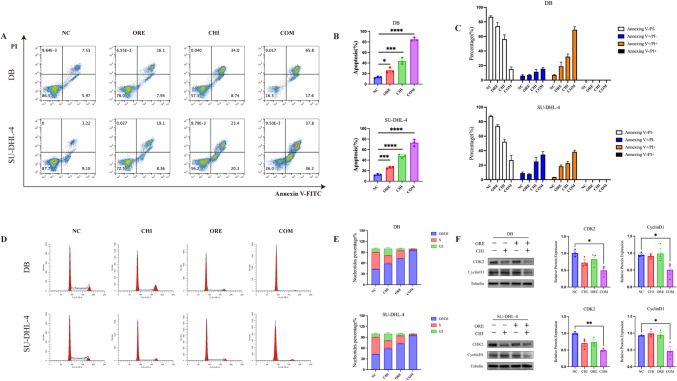
Effect of chidamide and orelabrutinib in DLBCL cell line apoptosis **A** and **B**. SU-DHL-4 cells or DB cells after 48 h of combined treatment with chidamide (2.*5* or 5 µmol/L) and orelabrutinib (60 or 70 µmol/L). The apoptosis rate of the combination group was significantly higher than that of the single-drug group. **C** In SU-DHL-4 and DB cells, the frequency of apoptotic cells (Annexin V-positive cells) significantly increased after exposure to chidamide and orelabrutinib. **D** The cell cycle distribution at 48 h was detected by flow cytometry. **E** Analysis of the cell cycle distribution results in **D**. **F** Effect on the expression of CDK2 and Cyclin D1 proteins in DLBCL cells. Quantitative analysis of the western blot results in CDK2 and Cyclin D1 proteins. Tubulin was used as internal control. *NC* negative control, *ORE* orelabrutinib, *CHI* chidamide, *COM* combination group, *PI*: propidium iodide, *FITC* fluorescein isothiocyanate (*P < 0.05, **P < 0.01, ***P < 0.001, ****P < 0.0001). Data are presented as the mean ± SEM

To further investigate the synergistic effects of combination therapy, we first examined cellular apoptosis after administering the combined drugs. As shown in Fig. 3A–C, SU-DHL-4 cells were treated with 2.5 μmol/L chidamide and 60 μmol/L orelabrutinib for 48 h; DB cells were treated with 5 μmol/L chidamide and 70 μmol/L orelabrutinib for 48 h; and the negative control was treated with DMSO. Each drug induced apoptosis in SU-DHL-4 and DB cells, the effects of chidamide and orelabrutinib alone were significantly lower than when used in combination.

To further investigate the effects of chidamide and orelabrutinib on the cell cycle of DLBCL cells, and the cell cycle distribution was analyzed by flow cytometry. In SU-DHL-4 and DB cells, compared with the control group, the cell cycle was arrested in the G0/G1 phase and the percentage of S phase was decreased with each drug alone, and this effect was enhanced with drug combination, the difference was statistically significant (Fig. 3D and E). The combination group significantly reduced Cyclin D1 and CDK2 expression in DB and SU-DHL-4 cells (Fig. 3F). Finally, these findings revealed that the combination of chidamide and orelabrutinib significantly could inhibit DB and SU-DHL-4 cell growth.

### Effects of chidamide and orelabrutinib on the transcriptomes of multiple genes in DLBCL cells

**Fig. 4:**
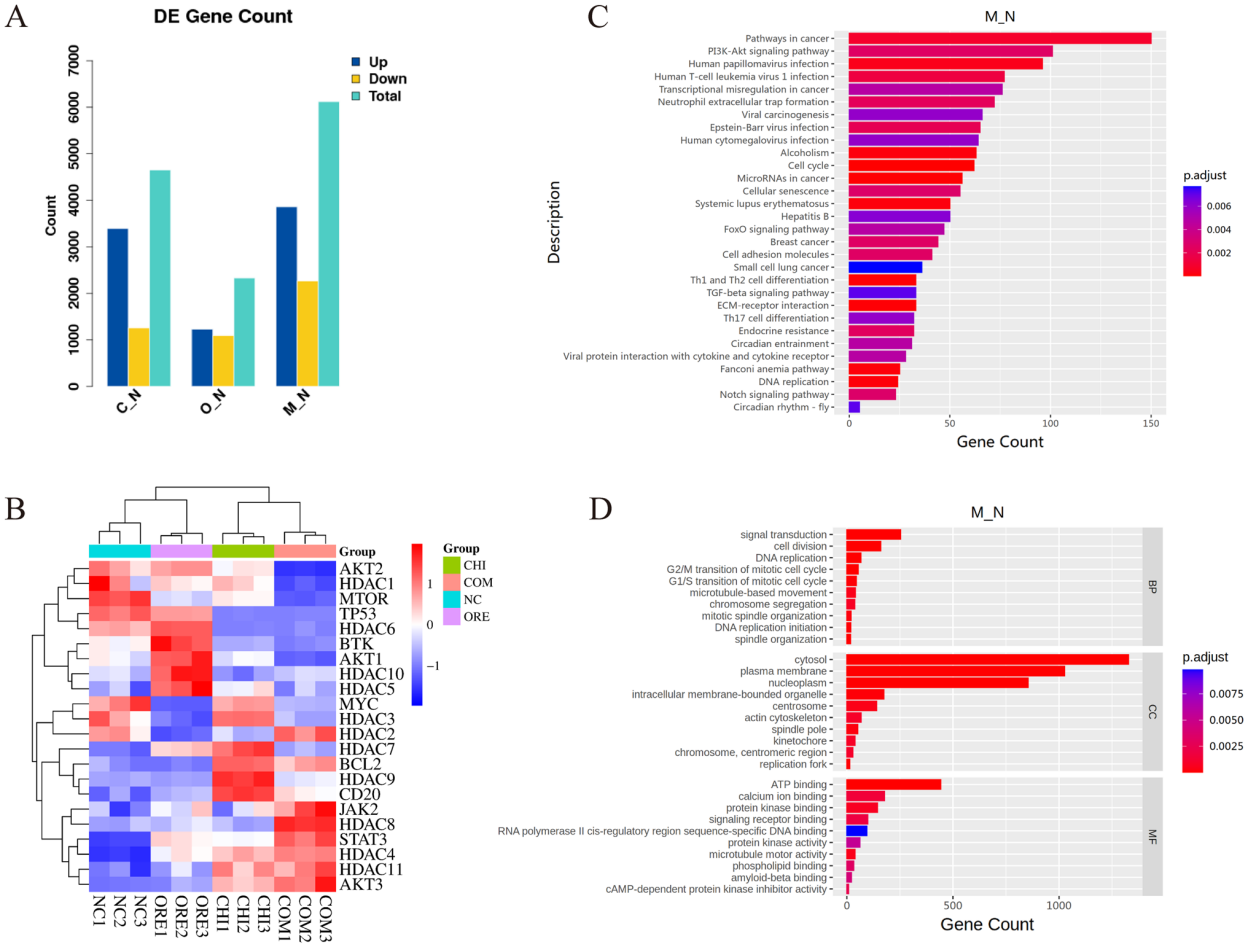
Effect of chidamide and orelabrutinib on the transcriptome of DLBCL cells. **A** The number of genes upregulated and downregulated in cell lines in the chidamide, orelabrutinib, and combination treatment groups compared with those in the negative control after 48 h of treatment with chidamide and/or orelabrutinib, abbreviated as C–N, O–N, and M–N. **B** Enrichment analysis of differentially expressed genes revealed the involved **C**. GO terms and **D**. KEGG pathways in the combination group. (*NC* negative control, *CHI* chidamide, *ORE* orelabrutinib, *COM* combination group)

Gene expression profiling was performed to investigate the mechanism of the synergistic antitumor activity of chidamide and orelabrutinib using RNA-Seq analysis. To better investigate the mechanism of the combination of chidamide and orelabrutinib on apoptosis and cell cycle arrest in DLBCL cells, we comprehensively analyzed transcriptional alterations in DB cells treated with 5 μmol/L chidamide and 70 μmol/L orelabrutinib for 48 h. The negative control group was treated with DMSO. We then performed genome-wide gene expression profiling (GEP; Fig. 4A); 4651 genes were regulated by chidamide, 2327 by orelabrutinib, and 6120 by the drugs in combination (logFC = 1, padj < 0.05).

Subsequently, we examined the transcription levels of signaling pathway genes, revealing that the mRNA levels of MYC, TP53, HDAC1, HDAC3, HDAC5, HDAC6, HDAC7, HDAC9, and HDAC10 were downregulated in the combined treatment group. Transmembrane 4-domain A1 (MS4A1, CD20), HDAC2, HDAC4, HDAC8, HDAC11, and BCL2 mRNA levels were upregulated in the combined treatment group. It is noteworthy that chidamide monotherapy could significantly upregulate the mRNA level of CD20 compared with the other groups in DLBCL cells (Fig. 4B).

GO and KEGG analyses showed that chidamide combined with orelabrutinib affected numerous crucial biological processes, including DNA replication, apoptosis, the cell cycle, and the PI3K/AKT/mTOR, MAPK, p53, B-cell receptor signaling pathways (Fig. 4C and D). Consistent with the apoptosis and cell cycle results, chidamide combined with orelabrutinib further promoted apoptosis and inhibited the aforementioned signaling pathways related to proliferation and differentiation.

### Effects of chidamide and orelabrutinib on ROS production and mitochondrial function in DLBCL cells

**Fig. 5:**
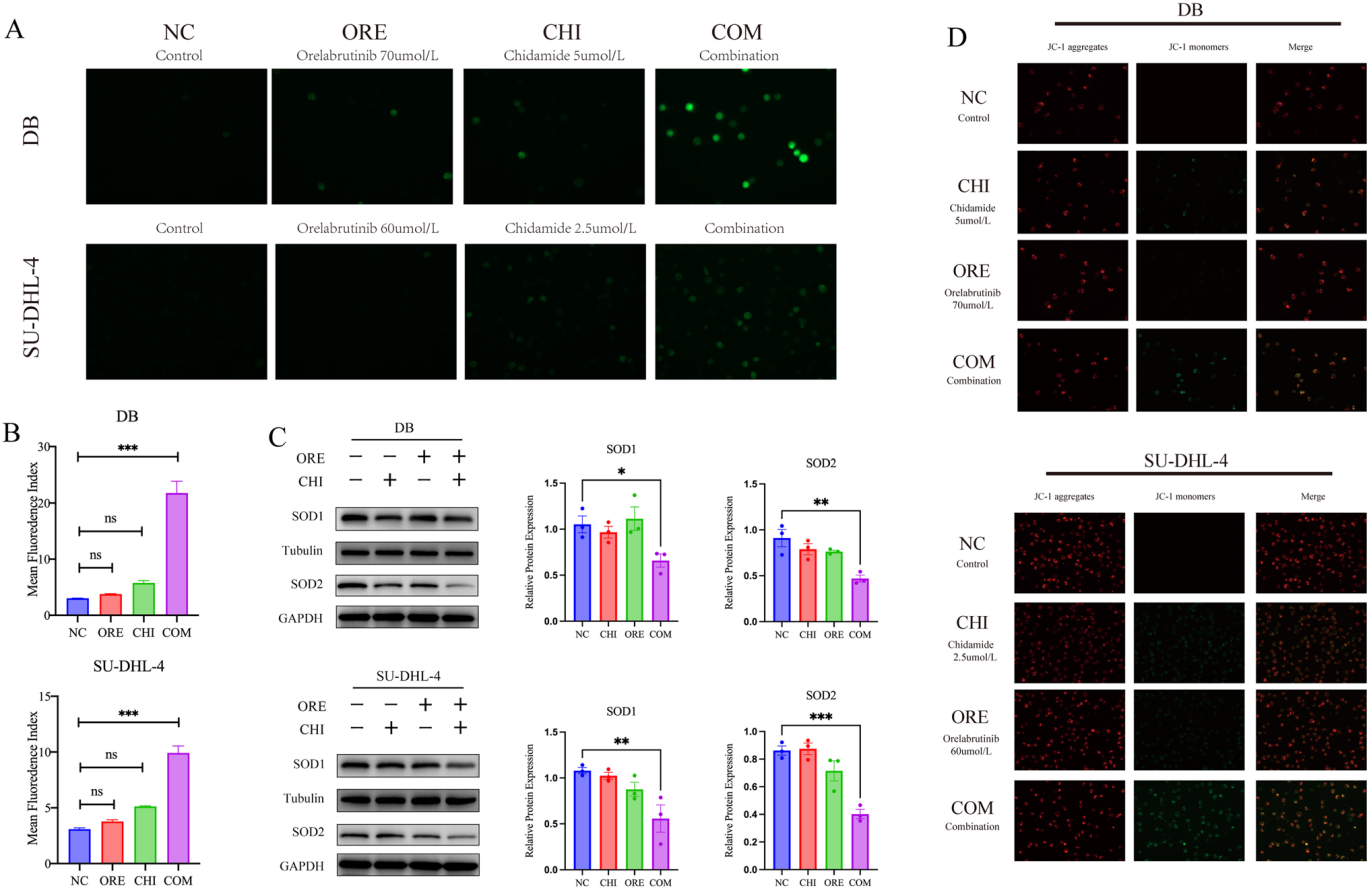
**A**, **B** ROS levels by DCFH-DA staining in SU-DHL-4 cells or DB cells treated with chidamide (2.5 or 5 µmol/L) and orelabrutinib (60 or 70 µmol/L) in combination for 48 h and fluorescence images were captured. **C** Effect on the expression of SOD1 and SOD2 proteins in DLBCL cells. Quantitative analysis of the western blot results in SOD1 and SOD2 proteins. GAPDH and Tubulin were used as internal controls. **D** The mitochondrial membrane potential changed in SU-DHL-4 cells or DB cells treated with chidamide (2.5 or 5 µmol/L) and orelabrutinib (60 or 70 µmol/L) for 48 h. Red fluorescence shows the JC-1 aggregates of the mitochondrial matrix, green fluorescence represents the JC-1 monomer of the mitochondrial matrix, and merge is the fusion of the two pictures. The data were expressed as means ± SEM (*n* = 3). (*P < 0.05, **P < 0.01, ***P < 0.001). (*NC* normal control group, *CHI* chidamide, *ORE* orelabrutinib, *COM* combination group)

The amount of ROS in cells was then quantified using the DCFH-DA staining technique. As shown in Fig. 5A and B, the green fluorescence intensity (DCFH-DA) was significantly increased upon treatment with chidamide and orelabrutinib in the combination treatment group compared with that in the control group. The combined group induced excessive ROS production in mitochondria, leading to oxidative stress and mitochondrial dysfunction. We used western blot to assess SOD1 and SOD2 protein expression levels to understand how oxidative stress is produced. The combined group significantly reduced SOD1 and SOD2 protein levels (Fig. 5C), indicating an escalation in oxidative stress levels during the cell death induced by the combined group. Excessive ROS production can promote apoptosis.

The JC-1 assay showed that the ratio of JC-1 aggregates to JC-1 monomers fluorescence intensity was significantly decreased in the chidamide and orelabrutinib combination treatment group compared with that in the control group, and the JC-1 fluorescence changed from red to green (Fig. 5D). This finding suggests that the intracellular mitochondrial membrane potential changes, initiating cell death.

### Effects of chidamide and orelabrutinib on c-Myc, BCL2, TP53, and other apoptosis proteins in DLBCL cells

**Fig. 6:**
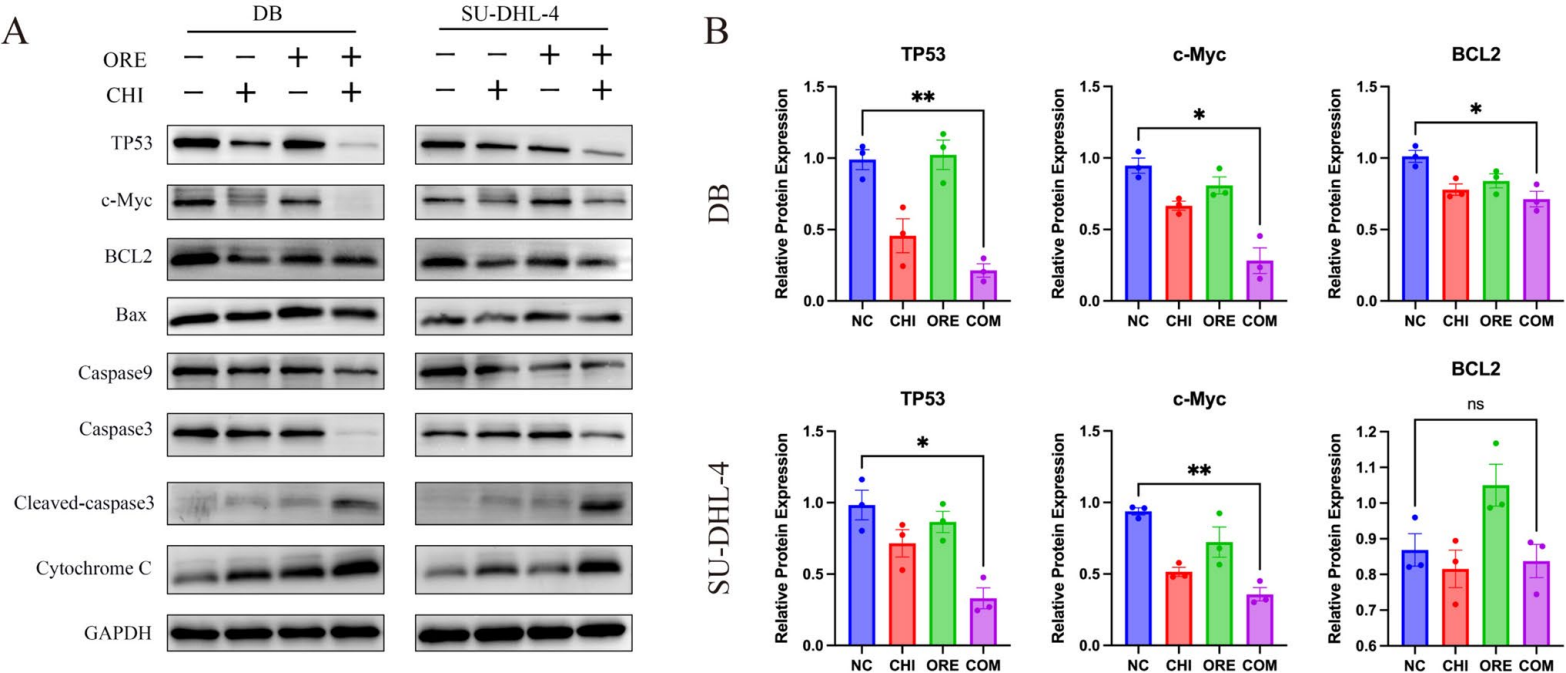
A. SU-DHL-4 cells or DB cells treated with chidamide (2.5 or 5 µmol/L) and orelabrutinib (60 or 70 µmol/L) for 48 h. Western blot was used to measure the expression of c-Myc, TP53, and apoptotic proteins. B. Quantitative analysis of the western blot results in A. Image software was used to analyze the gray value changes in c-Myc, TP53, and BCL2. GAPDH was used as the internal control. Data are presented as the mean ± SEM. (*P < 0.05, **P < 0.01). (*NC* normal control group, *CHI* chidamide, *ORE* orelabrutinib, *COM* combination group)

SU-DHL-4 and DB cells were treated with chidamide and orelabrutinib for 48 h to determine the response of c-Myc, BCL2, and TP53 protein levels to both drugs. As shown in Fig. 6A and B, we observed a consistent reduction in c-Myc protein levels in the two cell lines evaluated. This finding suggests that the drug combination group inhibited c-Myc regardless of c-Myc rearrangement status. Furthermore, the combination of chidamide and orelabrutinib significantly reduced TP53 protein levels, suggesting that the combination group inhibits TP53 regardless of whether TP53 is mutated. The BCL2 protein level was significantly decreased in DB cells in the combination group but not in the combination-treated SU-DHL-4 cells. The expression levels of other apoptosis-related proteins, Caspase3 and Caspase9, were also decreased, and those of Cleaved-caspase3 and Cytochrome C (Cyto-C) were increased. Therefore, we hypothesize that combining chidamide and orelabrutinib could improve DLBCL patient outcomes.

### Effects of chidamide and orelabrutinib on the autophagy-related proteins LC3B and P62 in DB and SU-DHL-4 cells

**Fig. 7:**
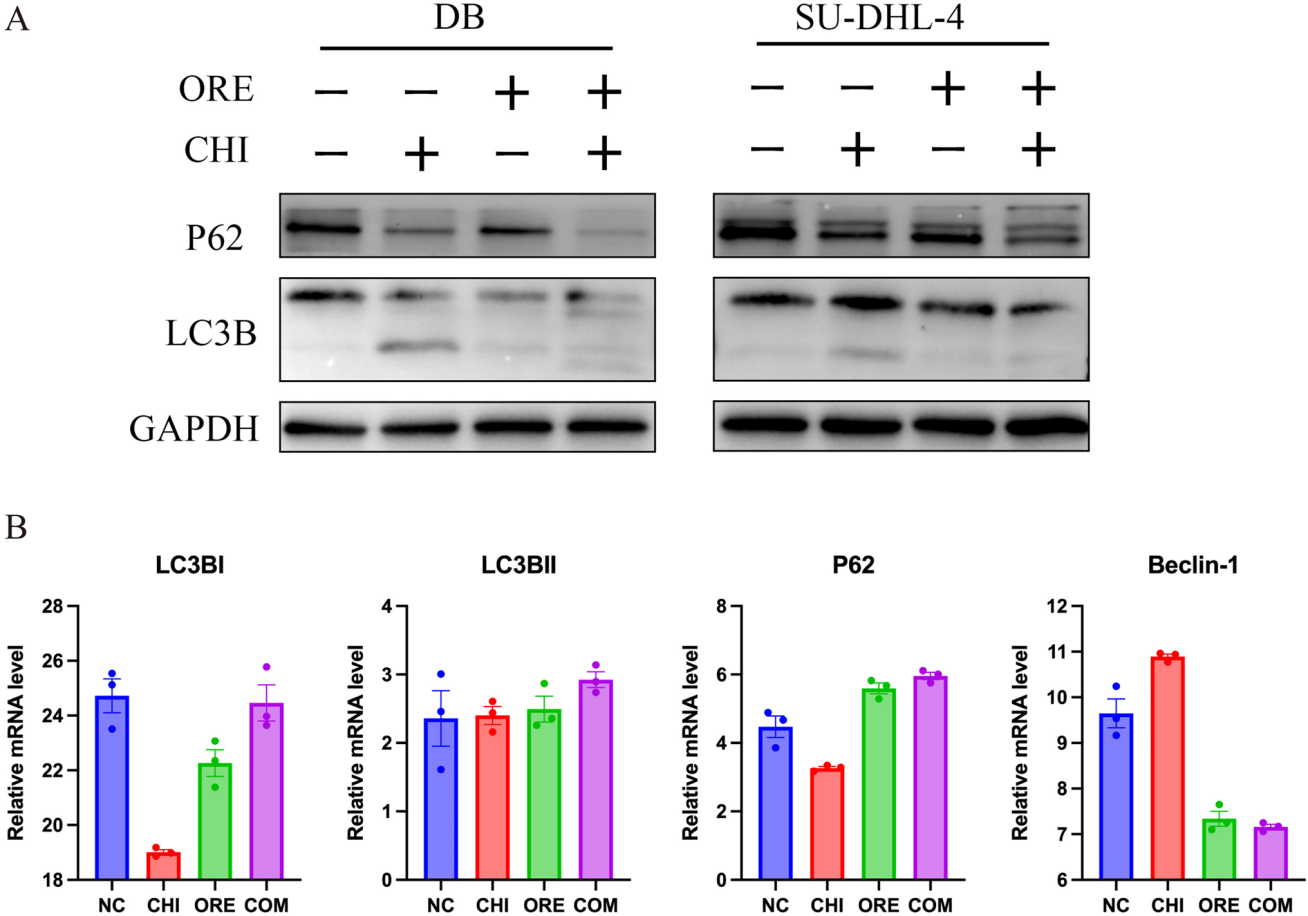
Effect on the expression of autophagy-related proteins in DLBCL cells. **A** Western blot was used to measure the effect of the intervention on the expression of autophagy-related proteins in SU-DHL-4 and DB cells over 48 h. **B** Statistical analysis of RNA-seq values for LC3B, P62, and Beclin-1 in DB cell. All data are expressed as the mean ± SEM (*n* = 3)

Increasing evidence indicates that excessive autophagy eventually leads to autophagic tumor cell death. Here, western blot and RNA-seq analyses showed (Fig. 7A and B) that chidamide upregulated the protein and mRNA expression levels of LC3BII and downregulated the expression of P62 in DLBCL cells. The mRNA levels of LC3I and Beclin-1 were also downregulated. LC3B and P62 are two key autophagy-related proteins. This finding confirmed that chidamide activated autophagy in DLBCL cells, consistent with previous findings. LC3BII was not upregulated in the orelabrutinib and combined treatment groups, suggesting that orelabrutinib may act as an autophagy inhibitor and block the regulation of autophagy.

### Chidamide synergizes with orelabrutinib and inhibits the growth of DLBCL cells by interfering with the PI3K/AKT/mTOR signaling pathway

**Fig. 8:**
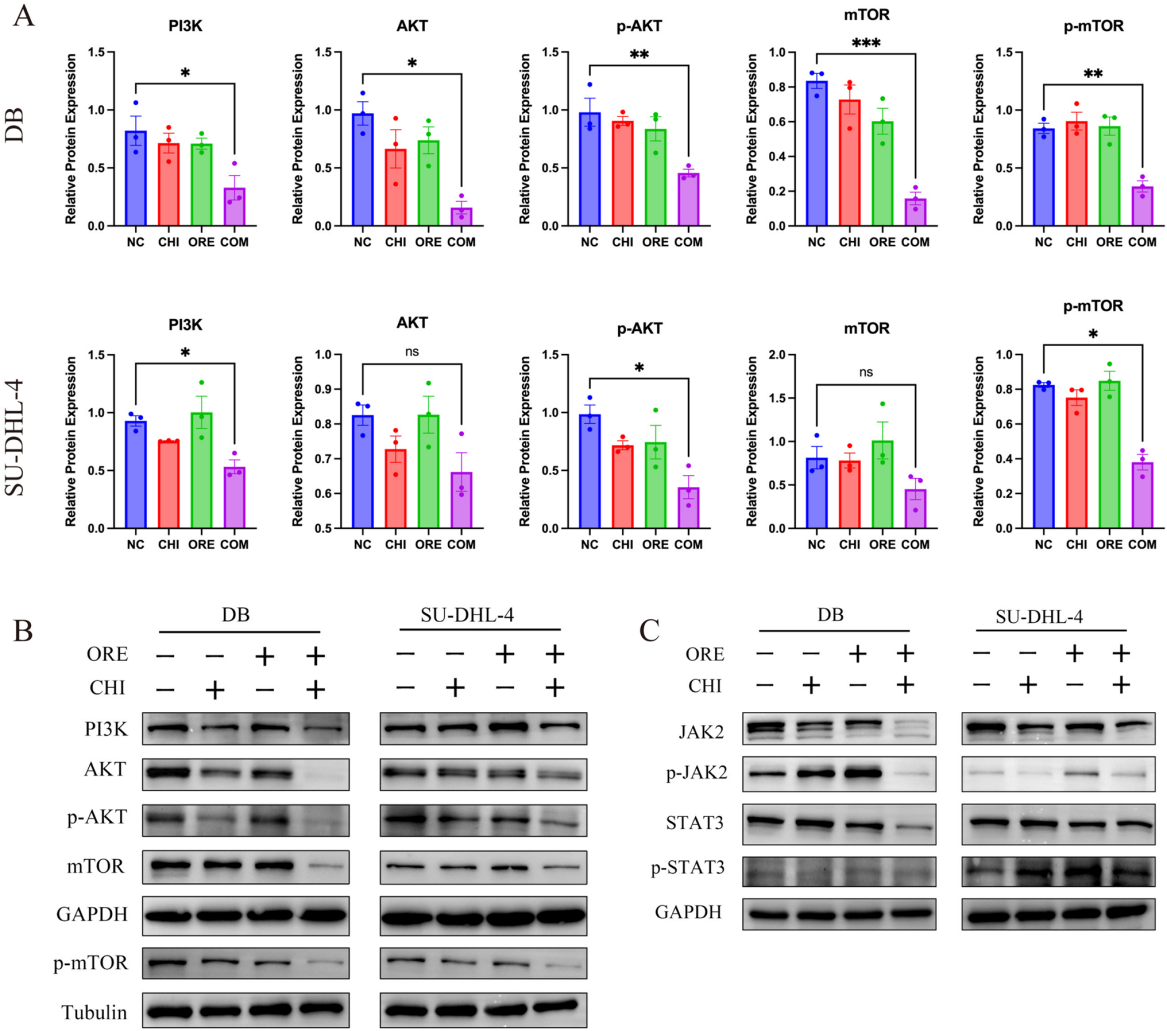
Effects of chidamide and orelabrutinib on the expression of PI3K/AKT/mTOR pathway-related proteins in SU-DHL-4 and DB cells. **A** Western blot of PI3K/AKT and mTOR signaling proteins in DB and SU-DHL-4 cells. **B** Statistical analysis of relative gray values of protein (PI3K (p85), AKT, p-AKT, mTOR, p-mTOR) bands (*n* = 3). GAPDH and Tubulin were used as internal controls. Data are presented as the mean ± SEM. (*P < 0.05, **P < 0.01, ***P < 0.001). (*NC* normal control group, *CHI* chidamide, *ORE* orelabrutinib, *COM* combination group)

**Fig. 9:**
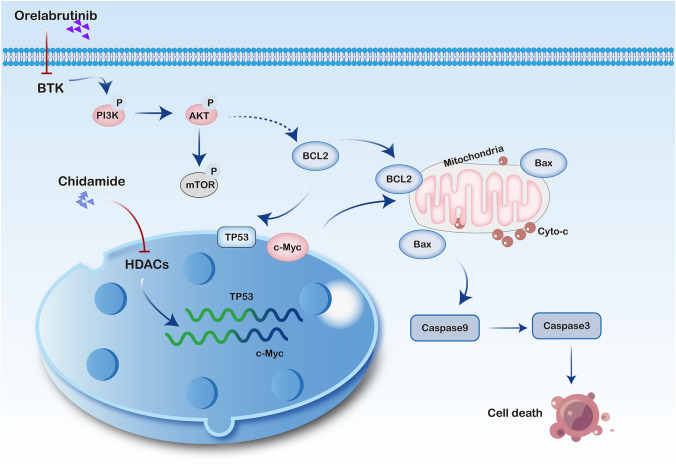
Mechanism of chidamide and orelabrutinib treatment. *HDACs* Histone deacetylases, *BTK* Bruton’s tyrosine kinase, *Cyto-C* Cytochrome C

To investigate the molecular mechanisms of chidamide and orelabrutinib in DLBCL cell lines, we next performed western blots to identify changes in different DLBCL cell lines after combined treatment at the protein level. The activity of the PI3K/AKT pathway and its downstream target mTOR were measured by western blot after 48 h of intervention in DLBCL cells. As shown in Fig. 8A and B, treatment of SU-DHL-4 cells or DB cells for 48 h inhibited the expression levels of PI3K (p85), p-AKT, and p-mTOR proteins compared with those in the control group (P < 0.05). There was no significant difference in the proteins on the JAK2 / STAT3 pathway (Fig. 8C). These findings indicated that the drug combination could inhibit the expression of the PI3K/AKT/mTOR signaling pathway and further regulate downstream apoptosis-related proteins, such as BCL2 and Caspase3. These results indicate that chidamide and orelabrutinib combined with the PI3K/AKT/mTOR pathway (Fig. 9) promote apoptosis of DLBCL cells.

## Discussion

In this study, we found that combining chidamide and orelabrutinib could significantly enhance the antiproliferative effect of these drugs. Compusyn software analysis showed that the chidamide and orelabrutinib combination resulted in a CI value < 1, indicating that this combination synergistically inhibits the proliferation of DLBCL cells. This synergism was also reflected in the combination’s inhibitory effect on DLBCL cell proliferation, the downregulation of cell cycle-related and antiapoptotic proteins, and the upregulation of proapoptotic proteins and ROS level. The combination of chidamide and orelabrutinib substantially reduced p-AKT levels, consequently augmenting mTOR pathway suppression. This finding provides a plausible explanation for the synergistic antitumor effect observed with the concurrent administration of these two drugs. The synergistic antitumor capacity indicates that double inhibition of BTK and HDAC is a potential new approach for cancer treatment, and the combined drugs are a potential treatment strategy for DLBCL.

Chidamide has been employed in preclinical and clinical investigations in combination with several small molecule-targeted drugs, such as inhibitors targeting EZH2, XPO1, BCL2, and others (Wang et al. 2021; Y et al. 2022; Luo et al. 2022). Rituximab is a monoclonal antibody that specifically targets the CD20 antigen expressed on the surface of B lymphocytes. CD20 expression is a promising biomarker for evaluating the clinical response of DLBCL patients to combination therapy (Suzuki et al. 2012). R-CHOP immunotherapy is the standard treatment for DLBCL. Clinical trials, such as NCT04231448, have incorporated chidamide to enhance the therapeutic efficacy of R-CHOP. The synergistic interplay of chidamide and rituximab has been demonstrated in vitro and in vivo, revealing chidamide’s significant capacity to overcome rituximab-mediated CD20 downregulation while promoting its anticancer effects (Guan et al. 2020). These results align with our sequencing data, suggesting that chidamide can upregulate CD20 expression in DLBCL.

Our bioinformatics analysis revealed elevated expression of epigenetic regulator genes, including HDAC and BTK, in lymphoma tissues. Moreover, BTK expression correlated with BCL2 expression, and BCL2, TP53, BTK, and HDAC genes exhibited a higher mutation rate in DLBCL tissues. The BCR signaling pathway plays a pivotal role in the pathogenesis of malignant B-cell lymphomas, including DLBCL. BTK, the most crucial kinase in the BCR pathway, is a prime target for inhibiting malignant B cell proliferation. BTK inhibitors are clinically employed as targeted therapies for DLBCL, downregulating the BCR signaling pathway (Advani et al. 2013; Ondrisova and Mraz 2020; Nakhoda et al. 2023). Compared with ibrutinib, orelabrutinib, a potent second-generation irreversible BTK inhibitor, exhibits higher selectivity and fewer inhibitory effects on other kinases. Furthermore, the use of BTK inhibitors as monotherapy is prone to resistance development. Combining therapies and the early implementation of targeted treatments can potentially alter the treatment paradigm for tumors, reducing the occurrence of BTK resistance (Ondrisova and Mraz 2020; Nakhoda et al. 2023). The PI3K/AKT/mTOR signaling pathway plays a significant role in normal physiological processes and in the development and progression of various diseases, including cancer (Porta et al. 2014). This pathway is also one of the most common pathways targeted for inactivation in tumors (Chia et al. 2015; Hoxhaj and Manning 2020; Harsha et al. 2020). In normal physiology, PI3K/AKT/mTOR signaling pathway regulates cellular activities, such as cell growth, cell cycle progression, and cell metabolism, through its downstream effectors 4EBP1 (4E binding protein) and S6K1 (S6 kinase beta1) (Shaw and Cantley 2006). In pathological conditions, aberrant PI3K/AKT/mTOR pathway activation disrupts cellular proliferation, ultimately leading to accelerated cell growth, enhanced angiogenesis, and increased drug resistance (Verret et al. 2019). The PI3K/AKT/mTOR signaling pathway plays a significant role in B-cell growth and development (Majchrzak et al. 2014), and in lymphomas, this pathway exhibits heightened activity. Dysregulation of this pathway is common in various cancers, with activated PI3K/AKT signaling associated with multiple processes, including the induction of tumor cell proliferation, inhibition of apoptosis, and promotion of invasion and metastasis (Yang et al. 2019). Aberrant activation of the PI3K/AKT/mTOR pathway has been observed in critical subgroups of DLBCL samples, driven by chronic BCR signaling activation or loss of expression of phosphatases and PTEN (Xu et al. 2023). The combination of orelabrutinib and venetoclax synergistically induces DHL cell death by modulating the PI3K/AKT and P38/MAPK pathways, inhibiting cell proliferation, and inducing cell cycle arrest (Pan et al. 2023). Our sequencing results highlight the PI3K/AKT and mTOR signaling pathways as potential targets in DLBCL. Similarly, the combination of chidamide and orelabrutinib downregulates the protein levels of PI3K and p-AKT, intensifying the inhibitory effect on the mTOR pathway. This effect further modulates proteins such as c-Myc, BCL2, and TP53, ultimately promoting DLBCL cell apoptosis.

In this study, SU-DHL-4 cells were used as a model representing DEL cells with WT-TP53, while DB cells served as a model representing DHL cells with MYC/BCL2 rearrangements and MUT-TP53. Both cell lines belong to the germinal center B-cell-like (GCB) subtype. Our study results demonstrate that the combination of chidamide and orelabrutinib exhibits a synergistic effect in comparison with the effect obtained when used as single-agent treatments. This combination therapy further suppresses the proliferation of DLBCL cell lines, induces cell cycle arrest in the G0/G1 phase, and promotes apoptosis in the majority of treated cells. The drug-induced apoptotic mechanism primarily involves epigenetic changes, silencing of the oncogene MYC, and induction of intrinsic apoptotic pathways. Our research establishes the synergistic antitumor efficacy of the combination of the epigenetic modulator chidamide and the BTK inhibitor orelabrutinib in DLBCL. This synergy is also evident in DHL cells with MYC/BCL2 rearrangements. The combination of chidamide and orelabrutinib concurrently regulates epigenetic alterations and affects the expression of genes such as c-Myc, TP53, and BCL2, achieving highly targeted and efficient precision therapy.

In summary, our research demonstrates the synergistic antitumor effect of chidamide and orelabrutinib combination therapy. This synergy is primarily characterized by further downregulation of PI3K/AKT/mTOR pathway activity, subsequently modulating the expression levels of cell cycle and apoptosis-related proteins, inducing cell cycle arrest and apoptosis. Our study findings provide a theoretical basis for the clinical implementation of chidamide and orelabrutinib combination therapy for DLBCL.

### Supplementary Information

Below is the link to the electronic supplementary material.

Supplementary file1 (DOCX 37 KB)

## Data Availability

The datasets used and analyzed during the current study are available from the corresponding author, Shaoling Wu, upon reasonable request.
