# Supplementary material for: Chidamide and orelabrutinib synergistically induce cell cycle arrest and apoptosis in diffuse large B-cell lymphoma by regulating the PI3K/AKT/mTOR pathway
Source: J Cancer Res Clin Oncol. 2024 Feb 21;150(2):98. doi: 10.1007/s00432-024-05615-7 (PMC10881688; doi:10.1007/s00432-024-05615-7)
Supplement: Supplementary file 1 — Supplementary file1 (DOCX 37 KB) [file 432_2024_5615_MOESM1_ESM.docx]

**Supplementary figure that would include dose effect curve and fractional effect**

| **DB** | | | |
| --- | --- | --- | --- |
| dose effect | driplot combo | iso combo | median effect |
| ***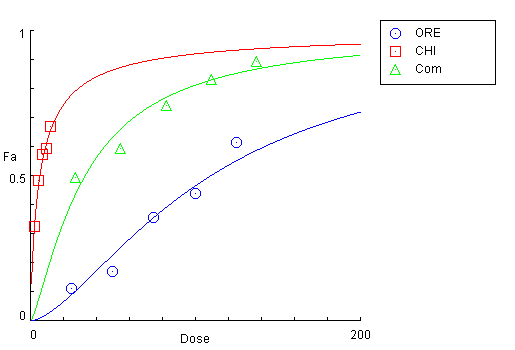*** | ***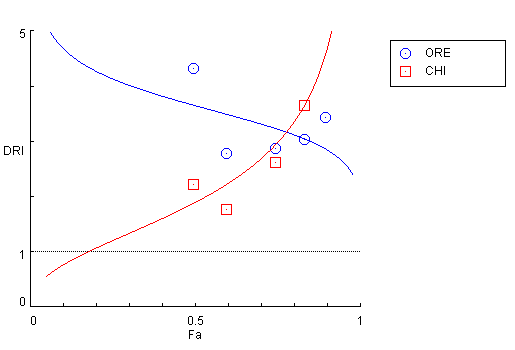*** | ***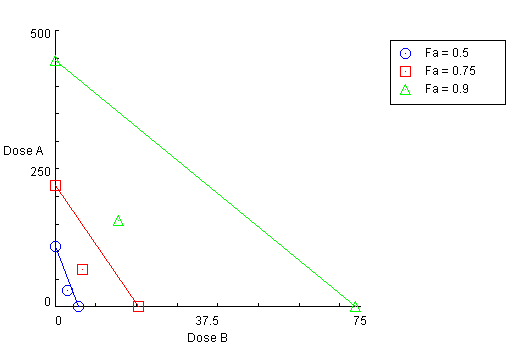*** | ***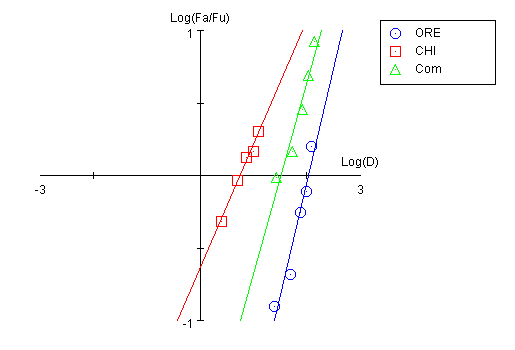*** |
| **SU-DHL-4** | | | |
| dose effect | driplot combo | iso combo | median effect |
| ***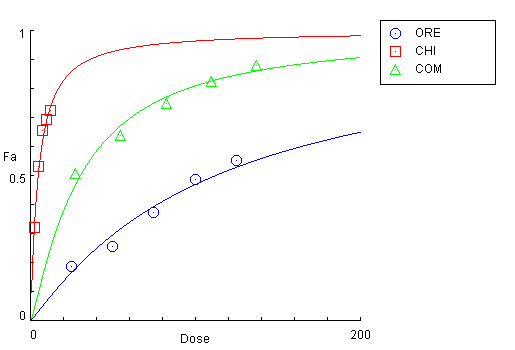*** | ***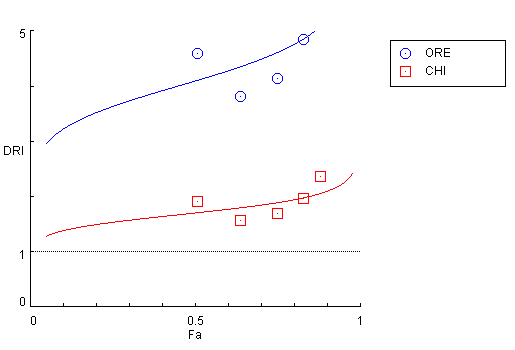*** | ***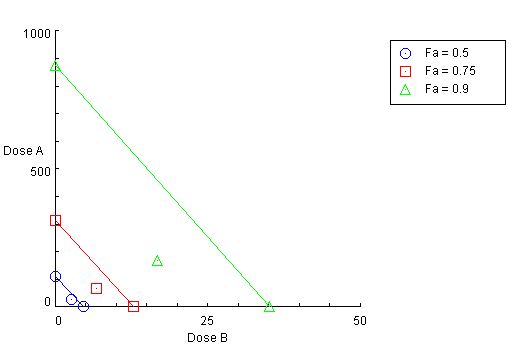*** | ***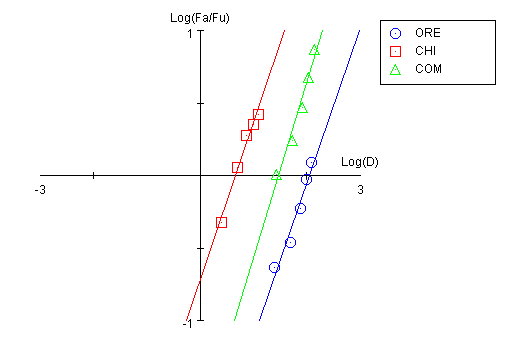*** |
